# Transplanting Cells for Spinal Cord Repair: Who, What, When, Where and Why?

**DOI:** 10.1177/0963689718824097

**Published:** 2019-01-18

**Authors:** Lyandysha V. Zholudeva, Michael A. Lane

**Affiliations:** 1 Department of Neurobiology and Anatomy, College of Medicine, Drexel University, Philadelphia, PA, USA; 2 The Spinal Cord Research Center, College of Medicine, Drexel University, Philadelphia, PA, USA

**Keywords:** cell transplantation, spinal cord injury, neural progenitor, interneuron

## Abstract

Cellular transplantation for repair of the injured spinal cord has a rich history with strategies focused on neuroprotection, immunomodulation, and neural reconstruction. The goal of the present review is to provide a concise overview and discussion of five key themes that have become important considerations for rebuilding functional neural networks. The questions raised include: (i) who are the donor cells selected for transplantation, (ii) what is the intended target for repair, (iii) when is the optimal time for transplantation, (iv) where should the cells be delivered, and lastly (v) why does cell transplantation remain an attractive candidate for promoting neural repair after injury? Recent developments in neurobiology and engineering now enable us to start addressing these questions with multidisciplinary expertise and methods.

## Introduction

**Fig. 1.**
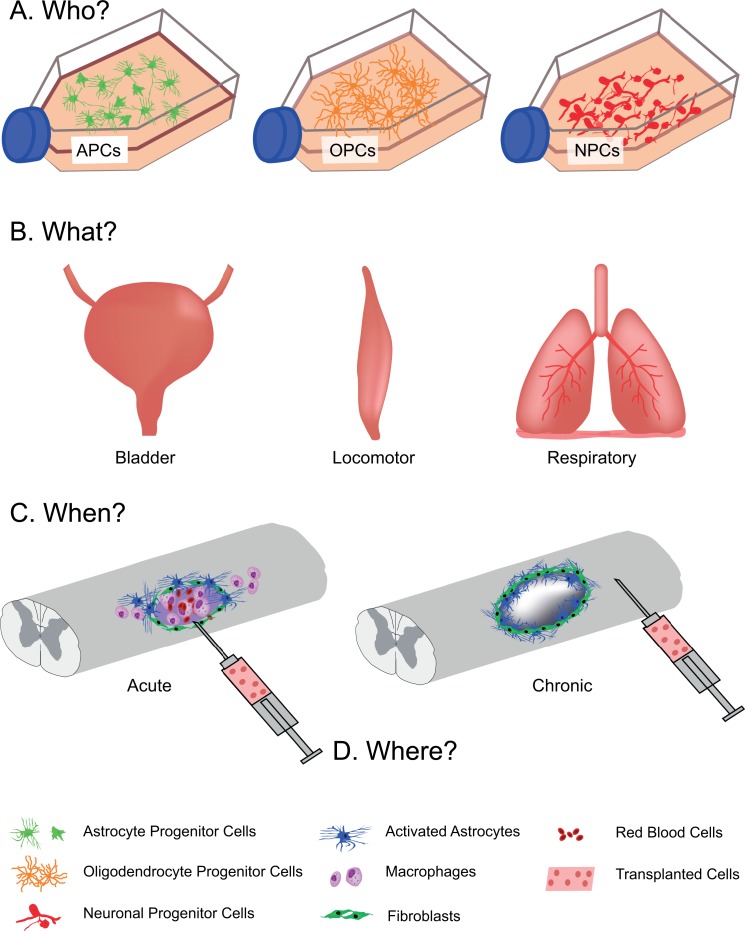
Transplanting for spinal cord injury. (A) Various cellular phenotypes can be cultured for cell transplantation after spinal cord injury. The cellular phenotype used will be dependent upon (B) what target system is being treated, as well as (C) when the cells are delivered, whether acutely (left) or chronically (right) after injury. Timing of transplantation will also influence the location of the injection (D), where in some cases, cells will be injected at the lesion epicenter (left) or distant from lesion site (right).

Advances in stem cell biology and cellular engineering have paved the way to a new era of cell transplantation. Several decades of pre-clinical and clinical research have shown that developing neural tissues, or the neural precursor cells that can be derived from them, can be transplanted into the injured central nervous system, integrate with surrounding host tissue, and promote both anatomical repair and functional improvement. From this research, it has become clear that donor tissues contain a highly heterogeneous population of neural phenotypes. The focus of this discussion will be on the use of neural cells for treatment of spinal cord injury (SCI). Tissues isolated from the developing spinal cord are inherently rich with the essential building blocks for repair: neuronal precursors (predominantly spinal interneurons; SpINs), glial precursors (astrocytic, oligodendroglial), vascular endothelial cells, microglia, and extracellular matrix^1–4^. This is consistent among transplants of either tissue blocks^5,6^ or freshly prepared, mechanically dissociated cell suspensions^7,8^. Chemically dissociating and culturing this developing tissue yields a more selected population of neuronal and glial restricted progenitors (SpINs, astrocytes, and oligodendrocytes). Until recently, the ability to characterize the phenotype of these donor cells has been elusive. Yet identifying and selecting specific donor cells is becoming crucial for effective treatment of the injured nervous system. Rebuilding functional neuronal networks within the injured spinal cord with transplanted cells will require donor neuronal elements that are capable of appropriate network formation and function. This has led to the notion of Who, What, When, Where and Why (Fig. 1)? *Who* are the donor cells being used for transplantation (e.g., is the neuronal phenotype defined), and are they used alone or in combination with other cells (e.g., neurons with glia)? *What* is the target organ (e.g., brain or spinal cord) and target network (e.g., hindlimb locomotor, respiratory, or sensory) for repair? *When* are the donor cells being transplanted (e.g., acutely vs. chronically) and what is the internal milieu of the injured nervous system like at that time? *Where* should donor cells be transplanted (e.g., at the lesion site or distant)? *Why* transplant cells for repair?

### *Who* are the Donor Cells that can be used to Treat the Injured Spinal Cord?

The focus of the present review is on transplantation of neural precursor cells (NPCs)—the cells within and cultured from developing neural tissues. Our increasing understanding of these spinal cord-derived neural elements and how they can contribute to repair guides us toward tailoring cell therapies for treating SCI. Some discussion will also include stem cell-derived NPCs, studies with which have often been built upon the knowledge gained from spinal cord-derived cells. With a growing appreciation for the range of neuronal and glial phenotypes that exist within the normal and developing spinal cord, those seeking to transplant NPCs have begun assessing donor cell phenotype more rigorously. These experiments began by using tissue obtained directly from the developing embryonic spinal cord. While often referred to as “fetal” tissue or cells, the term is typically used to describe cells derived from developmental tissue beyond the blastocyst stage (i.e., more mature than embryonic stem cells) without distinction between embryonic and fetal stages of development. This may be a misnomer, especially when applied to rodent systems that have a relatively short fetal stage (embryonic day (E) 17–21 in rats).

Early studies by Reier et al.^5^ demonstrated that donor cells harvested directly from the developing spinal cord (tissue blocks or mechanically dissociated only) provided a vastly heterogeneous population of cells for transplantation into the injured adult spinal cord. This has since been replicated independently by our research team^8^ and others^4,9^. They also had the capacity to retain their long-term phenotype, yielding mature spinal cord morphology^6,10,11^, and they become integrated with host neurons^6,12–14^. These cells were also capable of modifying the internal milieu of the surrounding injured spinal cord, making it more permissive for repair^15–17^. So, who are each of the donor cells that contribute to this repair?

#### Neuronal precursors

Neuronal precursors can be identified by molecular markers such as cadherins (ENCAM), neurofilaments, and microtubules (beta-3 tubulin, microtubule associated proteins). A vast range of transcription factors have also been characterized, enabling the histological identification of specific neuronal subtypes^18^. Advances in molecular genetics and developmental biology have elucidated specific SpIN subtypes via their transcriptional factor profiles^18,19^, which are present at the age identified to result in optimal cell survival after transplantation (E13.5–14 in rat^5^, E12.5 in mouse). As a result, we have a better understanding of the development of specific SpIN precursors and their roles in motor and sensory neural circuits. These circuits contain an intricate balance of excitatory, inhibitory, and neuromodulatory SpINs. Understanding this balance in the normal spinal cord, and how neuroplasticity after injury may change this balance, will help predict which donor cell populations should be used for repair. It should be noted that spinal tissues dissected at this developmental stage (equivalent to E13–14 in rat) cut the axons of spinal (lower) motoneurons that have developed already, resulting in retrograde cell death. Accordingly, examples of spinal motoneurons within tissues isolated at this time are rare^20^.

While ventrally derived tissues comprise primarily glial progenitors and motor or pre-motor interneuronal precursors, dorsally derived tissues comprise mostly interneuronal precursors with sensory functions^18,19^. White et al.^6^ demonstrated differences between transplantation of dorsally and ventrally derived developing spinal cord tissues, when transplanted to repair phrenic (diaphragm) motor networks after cervical SCI. This study revealed that while ventrally derived tissue can be functionally beneficial, dorsally derived tissues may in fact limit the potential for motor recovery. With this in mind, recent studies have begun to focus on subsets of these SpINs for transplantation^21^, to assess which cells may be most effective for repair. The V2a SpINs have been a strong candidate for repair of motor networks^21–24^. In the uninjured spinal cord, it is a pre-motor, excitatory SpIN that projects ipsilaterally. Thus, if transplanted on the side of the injury, they should increase activity within otherwise denervated motor neurons ipsilateral to injury. The V2a SpINs have also been associated with spontaneous neuroplasticity, further supporting their potential as a therapeutic target^25,26^. In contrast, quite different cell types may be more effective at treating hyperreflexia, spasticity, and/or pain^27,28^. With advances in cellular engineering, work is underway to purify several populations of distinct SpINs for transplantation^29–32^.

Studies initiated three decades ago by the Rao^1–3,33–35^ and Fischer^4,36–43^ teams have revealed a great deal about the effects of culturing E13.5–14 spinal cord tissue prior to transplantation, and the refined the populations of neuronal and glial restricted progenitors (NRPs and GRPs, respectively) that result from the process. For example, the E14 rat spinal cord comprises approximately 5–10% of multipotent neuroepithelial cells (NEPs), 30% GRPs, and 60% NRPs^1^. Isolation and culture of these cells refines the donor populations to neuronal and glial progenitors (40%:60%, respectively)^1^ that have a capacity for self-renewal but restricted differentiation fate (i.e., only become neurons or glial cells)^4,38^. However, less is known about the neuronal subtype of these cell populations with culture and how this may change over time.

While this discussion has centered on transplantation of spinal neuron progenitors, brain and brainstem neurons have also been tested for repair of the injured spinal cord^44^. While early tissue transplant studies used developing brain and spinal cord sources, the latter was found to be most effective for spinal cord repair. However, brainstem-derived neurons with modulatory functions have been shown to be effective for improving function after SCI^27,45–47^. Transplanting embryonically derived neuromodulators (e.g., serotonergic cells derived from the developing brainstem^45–47^) at the lumbar level may facilitate recovery of locomotor circuits by activating preserved components of the central pattern generator. Brain-derived neural stem cell populations have also been used to treat the injured spinal cord^48–52^. These studies also confirmed that the surrounding host environment influences the fate of transplanted stem cells, which become both neuronal and glial populations.

While spinal motoneurons are not often identified in transplants of embryonically derived spinal tissues, it is possible to attain large diameter, cholinergic, putative motoneurons from spinal tissues obtained earlier^53,54^, or derived from stem cells. Donor motoneuron candidates survive transplantation into the adult spinal cord, and retain neuronal morphology, but growth and connectivity to peripheral targets seems to be a much greater challenge. In contrast, donor motoneuron candidates transplanted into peripheral nerve survived and served as a relay between surviving host motoneurons and their peripheral targets^55–57^. Ongoing work in this area will better ascertain how to work with this population of donor cells.

At present, there is a general understanding of the types of precursors that exist within the developing nervous system at ages used to source donor tissue. However, the effects of isolation and cell culture on these phenotypes, and how they may be affected by the injured adult spinal cord once transplanted, is less clearly defined. We recently showed that just two days of 2D or 3D-cell culture altered transcription factor expression^22^. Alternatively, stem cell-derived neuronal populations can be driven toward specific phenotypic fates, but whether these fates are retained after transplantation, or whether they achieve the appropriate long-term function, is a subject of ongoing investigation. With a growing use of biomaterials to support transplanted cells, another consideration is how the biomaterial may affect which neuronal phenotypes survive pre- or post-transplantation into the injured spinal cord. These considerations are not unique to donor neurons, as they may also influence survival and differentiation of transplanted glia.

#### Glial precursors

Glial precursors and subtypes can be identified by molecular markers such as surface gangliosides and receptors (i.e., A_2_B_5_, platelet-derived growth factor receptor alpha), intermediate filaments and cytoskeletal proteins (i.e., vimentin, glial fibrillary acidic protein), transcriptional markers (oligodendrocyte lineage transcription factors), and other binding proteins (i.e., ionized calcium binding adaptor molecule 1, Iba1). The developing rat spinal cord at E13–14 comprises astrocytes, oligodendrocytes (and their precursors), and microglia. Astrocytes during these development stages are supportive of neural growth and development, and can facilitate endogenous axonal regeneration^58–60^. However, transplantation of GRPs cultured from E13–14 rat spinal cord, into the injured adult spinal cord, has been shown to attenuate inhibitory aspects of the injury environment and enhance host axon growth^17,37,61–63^. Immunohistochemistry of transplanted GRPs *in vivo* suggests that most donor cells become both astrocytes and oligodendrocytes^62^. While both Type 1 (protoplasmic, A_2_B_5_-negative) and Type 2 (fibrous, A_2_B_5_-positive) astrocytes have been reported^2^, additional studies are required to assess A1 versus A2 phenotype of these cells^64^. Transplantation of mature astrocytes derived from GRPs *in vitro* also survive, migrate into the injured spinal cord, and improve sensory function post-SCI^62^. With a focus on respiratory networks after cervical SCI, Li et al.^65^ demonstrated improved functional outcome following transplantation of induced pluripotent stem cell (iPSC)-derived astrocytes.

GRPs can also give rise to oligodendrocytes, but the extent may be dependent on cell preparation. Jin et al.^62^ found that the majority of donor GRPs at the lesion and transplantation site are astrocytic (∼80%), while the numbers of astrocytes and oligodendrocytes more distant from the lesion site become more even. The migration of oligodendrocytes away from the lesion and toward host axons could be an indication of these donor cells migrating to myelinate axons. This regional difference in donor glia may reflect important influences that the injured host spinal cord has on differentiation and/or migration of donor cells, and raises the importance of defining *where* cells should be transplanted (see below). In contrast, Lu et al.^9^ found limited migration of donor glia away from the transplant epicenter when delivered with neuronal progenitors, and a larger proportion of donor oligodendrocytes (27% oligodendrocytes, 16% astrocytes).

Another cell source that has been used both pre-clinically and clinically is selected populations of oligodendrocyte precursor cells (OPCs). Using stem cell-derived OPCs, Keirstead et al.^66–70^ and others^71–74^ found that donor cells promote repair post-SCI, contribute to myelination, and improve functional outcome. These initial experiments led to the translational investigation of OPCs for treatment of SCI^74^, with clinical trials initiated by Geron, and more recently by Asterias.

While technically not of a neural lineage, donor microglia are found in transplants of developing spinal cord tissue. However, the contribution of these donor microglia to inflammation, survival, or development within donor tissue remains poorly defined. Chemically dissociating and culturing this donor tissue source, however, selects for astrocytic and oligodendroglial precursors and removes microglia. This is an example of the greater range of cell types that can be present in non-cultured donor tissue.

#### Other cell types

While cultured donor neural precursors are composed primarily of NRPs and GRPs (at a ratio of approximately 2:1^1^), developing neural tissues used for transplantation (not cultured) contain many other elements that may also contribute to neural repair. In addition to microglia, this donor tissue includes extracellular matrix and vascular endothelial cells, which support cell survival and growth through the expression of growth-supportive proteins (e.g., laminin, FGF, VEGF). Donor vascular cells may support growth by recapitulating neural development and providing a growth-supportive surface for axon growth cones. The presence of these components may also contribute to improved vascular repair seen with tissue transplantation, restoring its metabolic capacity^75^. With only limited effort made to assess their potential as donor substrates^76^, the focus has remained on neuronal and glial elements.

We now have the tools to identify, screen, and enrich for specific cell phenotypes destined for transplantation. With this in mind, the question of *what* the target system is for treatment becomes an important consideration (see below).

### *What* is the Intended Target?

As the phenotype of donor cells becomes more clearly defined, there is a need to reconsider *what the intended target for repair is*. Within the injured spinal cord, which neural network is the intended focus? While it would be ideal to transplant cells that are effective at repairing all circuits, donor neurons will have specific functions and may be effective for some circuits more than others (e.g., motor vs. sensory). While lower (spinal) motoneurons clearly target motor systems, there is a wide range of SpIN phenotypes with equally variable contributions to motor and sensory functions. As described above, White et al.^6^ reported differences when transplanting tissue derived from the dorsal versus ventral developing spinal cord (rich with unique neuronal progenitor phenotypes). With some evidence for cells of defined neuronal fate retaining the capacity to synaptically integrate with appropriate host circuitry^77^, a logical goal for network repair will be to harness neuronal cells that are not only found within the network being treated, but are also capable of driving recovery (e.g., contribute to endogenous neuroplasticity). Thus, identifying the target network and the normal components of that network are essential for optimal treatment with transplanted cells.

One cell that is of increasing interest as a target for SCI is the SpIN^19,78,79^. SpINs are known to not only contribute to control and modulation of function, but are also key neural elements in plasticity after SCI^79–81^. Recent pre-clinical studies are beginning to identify specific subsets of SpINs that play restorative roles after SCI. Within the phrenic motor circuit—which controls the diaphragm—a subset of excitatory cells known as “V2a” INs have now been shown to contribute to plasticity in pre-clinical models of amyotrophic lateral sclerosis^82^ and SCI^25^. Capitalizing on this finding, we recently demonstrated that transplantation of NPCs that were enriched with V2a cells resulted in improved phrenic motor recovery after cervical SCI^83^. In contrast, transplantation of inhibitory neurons may be better suited for treating pain and spasticity. Fandel et al.^27^ demonstrated improved recovery of bladder function and neuropathic pain in a mouse model of SCI, following transplantation of inhibitory neurons. Thus, it is possible that each system being treated will benefit from unique donor phenotype combinations.

However, these strategies assume that efficacy is best achieved either via donor cell–host circuit integration, or that donor cells can elicit necessary effects without synaptic integration (e.g., neurotransmitter release into transplanted area). Thus, the intended goal is to either “replace” relevant cells that are lost following injury, or provide new populations of cells that elicit pro-neuroplastic properties and enhance recovery. Alternatively, “by-stander” effects of donor cells (e.g., trophic factor or cytokine release) may be sufficient to achieve some recovery, as evident in work focused on transplanting donor cells that maintain their stemness even after transplantation^84^. In either case, the system being treated and the temporal, anatomical, biochemical, and functional changes within the compromised network, then raises the question: when is the optimal time for cell transplantation?

### *When* is the Optimal Time for Repair?

Timing of treatment following injury depends primarily on treatment goal (treating the acute vs. chronic pathophysiology), which also influences which donor cells would be optimal for repair. It is also a crucial consideration for cell transplantation strategies in general, as temporal changes in the host environment may affect donor cell survival, proliferation, and differentiation^7,8^ ^5^. Cell therapies have been used to neuroprotect the injured nervous system and limit tissue damage (e.g., transplanting immune regulatory cells such as glia), enhance spontaneous neuroplastic mechanisms, restore tissue continuity and provide a growth permissive substrate for axonal growth and repair, and replace lost neurochemical input (e.g., transplanting neuromodulatory cells such as serotonergic neurons). In general terms, treatments can be divided into those applied acutely, sub-acutely, and chronically. As outlined in Table 1, the cells used may change depending on timing post-injury, with important considerations for each condition. It should be noted that our current appreciation for these treatment times comes primarily from pre-clinical studies, and how these relate to treatment of human injuries remains less clearly defined.

**Table 1.** These Categories are Defined by Our Own Pre-Clinical Studies, and Others^51,90,92–96^. While There is Some Variability in These Defined Time-Windows (Likely Differences in Animal and Injury Models), the Characteristics Used to Define Them are Comparable (e.g. Chronic Injury is Typically When the Lesion Epicenter and Peri-Lesional Areas are Stable).

Acute	<48hrs
	Goal: Transplantation within the early stages post-injury (e.g. 24-48 hours) to target inflammation and promote neuroprotection, limit axonal retraction, and reduce secondary tissue damage.
	Cells used: Growth-supportive/permissive, anti-inflammatory, and pro-vascular cells. In addition, donor cells may be able to restore metabolic homeostasis, thus enhancing neuroprotection. Usually delivered to the lesion epi-center or peri-lesional area.
	Considerations and barriers to cell therapy: Donor cell survival may be limited as the pro-inflammatory internal milieu of the lesion epicenter is not conducive for survival at such early time post-injury. This may not be a concern, provided donor cells survive long enough acutely to exhibit the necessary effects (e.g. cells are transplanted with the intended purpose of secreting anti-inflammatory and/or neuroprotective factors).
	References: ^4,6,11,84–89^
Sub-acute*	48 h–4wks
	Goal: Facilitate repair during ongoing neuroplasticity and anatomical reorganization within the injured host spinal cord.
	Cells used: Growth-supportive/permissive (may modify the glial scar) cells, pro-vascular cells, and neurons capable of forming networks and integrating with host neurons—delivered to lesion epicenter or peri-lesion area. Also, neuromodulatory cells (e.g., serotonergic) which can be delivered distant to injury near denervated cells (e.g., lumbar spinal cord).
	Considerations and barriers to cell therapy: With ongoing anatomical and biochemical changes during this stage, care needs to be taken to not disrupt otherwise beneficial neuroplastic mechanisms. Potential disruption and/or inhibition of adaptive plasticity is the greatest barrier to cell transplantation at this time point injury. However, treatment during this stage when plasticity is ongoing may enable better, if not most optimal, growth and integration between donor and host.
	References: ^8,9,22,43,75,88,90^
Chronic	>4–12 weeks
	Goal: Cells that may facilitate delayed repair and contribute to additional plasticity. Modify the existing glial scar at the lesion site, and promote vascularization. While there has been some concern that the capacity for repair may be reduced at very late chronic stages, following a longer period of wound healing/scarring, there is mounting evidence to suggest that this “window” for treatment can be reopened to facilitate repair at even very late stages.
	Cells used: As described for the sub-acute stage.
	Considerations and barriers to cell therapy: Perhaps one of the most important considerations here is what defines the treatment time as “chronic”. “Early” chronic stages (about 4–12 weeks) have been described as “sub-chronic” or intermediate, with “chronic” referring to even later stages (>12 weeks). Anatomical and biochemical changes become more stable at around 4–6 weeks, with less spontaneous axonal sprouting and plasticity during this stage. Immunological events may still be ongoing within the injured spinal cord at this time, but become more stable 8–12 weeks post-injury. If transplantation requires axonal growth, the donor cells need to stimulate it, or an additional treatment may be required to do so. Directing host and donor growth may require activity-based therapies or neural stimulation (e.g.,^91^). The greatest barrier to cell therapy at the chronic timepoint is the state of the lesion itself. It is unknown if the potential for repair is reduced, whether the chronic state of the scar is no longer receptive for growth of donor or host cells and whether the benefits and effects of transplantation will be comparable to what has been shown at more acute stages. Clinically and logistically, transplantation at the acute stage post-injury can be performed in combination with other necessary surgeries (e.g., decompression). In contrast, surgery at the chronic stage may be more difficult once more extensive scarring has occurred and the chronically injured patient may not recover from a surgery as quickly.
	References: ^7,51,90,92^

* Despite being misleading (sub-acute would typically refer to events pre-acutely), this is a commonly used term in the field to refer to times soon after the early acute stages of injury.

While many pre-clinical studies first test treatment efficacy in acute or sub-acute models of SCI, there has been a pre-clinical and clinical push toward developing treatments for more chronic time-points, which will then benefit a much larger population. Pre-clinical studies have even begun assessing treatment efficacy in aged rats, more than a year post-injury^97^.

Clinical trials using cell therapies are also expanding their treatment window to target a wide population of people with SCI. As our understanding of the temporal changes within the injured spinal cord improves—at and distant from the injury site—the question becomes: where donor cells can and/or should be delivered to optimally improve outcome?

### *Where* Should Donor Cells be Delivered?

When deciding where to transplant cells to treat the injured spinal cord, one must consider the goal of transplantation and accordingly also the cells being used. For example, while oligodendrocytes might be transplanted into regions of primary demyelination, or those containing newly growing (unmyelinated) fibers, donor neurons capable of replacing damaged or dead neurons should be delivered into the lesion cavity. In contrast, donor neurons intended to provide neuromodulatory functions (e.g., serotonergic cells) may need to be delivered to denervated networks distant from the injury site. Once the location is established, the cytoarchitecture at that site needs to be defined. The location where donor cells are implanted can also affect their phenotype^98^, which likely impacts functional outcomes.

While cell transplantation by its very nature is invasive, the goal remains to be minimally invasive. Thus, delivery of cells to distant locations must take into account that the tissue—while perhaps denervated—is relatively intact. In contrast, delivery to the lesion epicenter is easier to justify, but then has different caveats. Yet, with most cell transplants designed to promote neural repair, the lesion site and/or the peri-lesional area is the most commonly used site for transplantation.

#### Transplanting into or below the injury site

If the lesion is penetrating (or a pre-clinical model of partial or complete section injury), there must be a mechanism to keep transplanted cells at the lesion site. Use of either donor tissue pieces, or combined delivery with a biomaterial^99–101^, has addressed this issue. Closed lesion sites associated with contusion or compression injuries result in cavitation in most species, providing an enclosed site for transplantation. However, the second issue associated with transplants into the lesion site is that it represents both a molecular and physical barrier to repair. Wound-healing processes post-injury result in scar formation that can prevent donor cell integration to some extent. Despite this, transplantation of neuronal and glial progenitors into the lesion epicenter has been shown to alleviate this, restoring tissue continuity and providing a bridge for axonal repair. There has been some suggestion that it is the donor glial progenitors that provide this modulatory effect on the scar^15,17^.

While many studies are focused on transplantation at the injury site, therapeutic effects can be achieved away from the injury site as well, especially if the intended goal is “by-stander” effects (trophic support, immunomodulation). Accordingly, donor cells can be transplanted wherever such effects are more warranted. Neuromodulation of motor output (e.g., serotonergic or catecholaminergic) can also be achieved with transplantation of these neuron types in the vicinity of target lower motoneurons that may be several spinal segments away from the injury. For example, the delivery of cells below the level of injury after a cervical or thoracic injury can still modulate function of the target cells for locomotion^45,46,102^ and respiration^47^. Intravenous injection of other donor cell populations (e.g. mesenchymal stem cells) has also been used to elicit by-stander effects that promote some functional improvement.

#### Intrathecal delivery of cells

While the vast majority of neural cell transplantation strategies (e.g., transplantation of neural progenitor cells) have utilized intraparenchymal injection of cells directly into the lesion site or immediately surrounding spared tissue, there are studies that have employed less invasive approaches such as intrathecal delivery of donor cells. Parenchymal injection of donor cells, albeit an efficient delivery method, is considered to be an invasive technique risking further damage to the injured spinal cord. Delivery of donor cells into the cerebrospinal fluid is a potential alternative and has been demonstrated as a feasible technique in pre-clinical studies using neural progenitors^103^ as well as bone marrow stromal cells^104–107^. In fact, previous work has demonstrated that both endogenous^108^ and transplanted NPCs^103,109^ can migrate to sites of pathology and contribute to anatomical repair of nervous tissue. The chemotaxis driving this migration can also be engineered. For instance, viral vectors can be used to induce trophic factor expression and promote migration of donor cells to relevant targets, as has been done with directing growth of donor axons^87^.

### *Why* Transplant Cells for Treatment of Spinal Cord Injury?

With an ever-expanding number of approaches being developed for treatment of the injured spinal cord, what is the benefit of using cell transplantation? First, most cell therapies in testing are a natural mix of cell types. For example, those derived from neural precursors contain neuronal and glial progenitors (at the very least). In that way, they can be seen as an endogenous (and biologically relevant) combinatorial strategy.

Second, cells are capable of a vast range of functions. Cells are biological elements capable of sensing, adapting, integrating with—and even modifying—their surrounding environment. Transplanted donor neurons are capable of synaptically integrating with host circuitry, and functionally contribute to restoring the communication once broken by a SCI. Donor glia (e.g., astrocytes) respond to the surrounding environment, but unlike host astrocytes, donor astrocytes appear to maintain a growth-supportive phenotype. These donor astrocytes not only modify the existing glial scar at an injury site, they also appear to guide host axons into the injury site and transplant. Like the endogenously proliferating host oligodendrocytes, donor oligodendrocytes not only support neurite myelination, but there is some suggestion that they also regulate inflammatory cascades. Lastly, donor endothelial cells serve as self-assembling biological scaffolds, coming together with host endothelial cells to restore tissue vascularity, while also supporting neurite outgrowth as seen in the developing nervous system.

While we begin to appreciate the vast capacity of cells available for transplantation and repair of the injured nervous system, the optimal approach will most likely be built on multidisciplinary strategies. For example, expertise in genetic, anatomical and molecular neural development, and neural network electrophysiology are crucial in identifying optimal cellular components for repair. Cellular engineering is critical to the generation of specific cell subtypes tailored for repair. Pre-clinical and clinical expertise in activity-based therapies (e.g., rehabilitation) and neuromodulation (neural interfacing) are essential in designing combinatorial strategies to help optimize transplant integration with host injured circuitry. Finally, communication between scientists, clinicians and patients/patient advocates is important for translational success: (i) therapies need to be developed to best meet medical needs, (ii) pre-clinical studies need to be conducted under consultation with neurosurgeons and clinicians that will eventually coordinate clinical trials, (iii) ongoing trials need to be conducted in parallel with pre-clinical research to continually refine and improve treatments, and (iv) translated treatments need to be reverse translated to re-assess treatment goals and ensure they continue to meet the needs of a changing patient population.

## Closing Remarks

Cells are likely capable of far more than we currently appreciate. Tailoring cell therapies for individuals is becoming a commonly sought goal. Once we establish the patient and neural network to be treated, and the intended post-injury time for treatment, then the appropriate donor cell can be engineered and transplanted into the injured spinal cord. One of the biggest limitations we currently face is that we are still trying to understand (i) *who* are the ideal cells for transplantation and *what* is the target for repair, (ii) what are the strengths and weaknesses of these cells for the target, (iii) how and *when* best to harness their strengths, and (iv) *when* and *where* to use them. Yet pre-clinical studies are now becoming more focused on these issues.

As appropriate donor cells are identified, logistical and ethical considerations become critical in then translating cell therapies. What is the original source of donor cell types used? Have they been obtained, prepared, and preserved adhering to established guidelines and legislature for global safety and ethics? Even if the optimal donor cell type(s) are identified, and are obtained safely and ethically, how are clinical trials conducted (e.g., blinded trials, controlled vs. open clinical trials)? For that matter, how often are cell therapies used to treat patients without rigorous pre-clinical and clinical testing (e.g., “investigational treatments” not yet approved, “compassionate use” of unapproved treatments in the critically ill, or “stem cell tourism”)? As new strategies develop for designing novel donor cell types, the regulations on clinical translation must be routinely re-evaluated to promote the safe and ethical use of these break-through technologies.

With an improved understanding of cell biology and neural development, neural phenotypes are becoming better classified and studies have begun focusing on use of more specific cell types for transplantation. Existing cellular engineering and stem cell biology methods now enable the development of purified populations of donor cells that can be genetically modified to alter phenotype and function. As we better understand the neural repair process, the temporal use of cell therapies will become more refined, and new cell types may be included in donor populations. As the techniques available to us improve and we better understand the donor cell populations, the coming years will bring exciting advances in cell therapies for spinal cord repair.
